# Superfast Capture of Iodine from Air, Water, and Organic Solvent by Potential Dithiocarbamate-Based Organic Polymer

**DOI:** 10.3390/ijms24021466

**Published:** 2023-01-11

**Authors:** Liya Thurakkal, Subba Rao Cheekatla, Mintu Porel

**Affiliations:** 1 Department of Chemistry, Indian Institute of Technology Palakkad, Palakkad 678557, India; 2 Environmental Sciences and Sustainable Engineering Center, Indian Institute of Technology Palakkad, Palakkad 678557, India

**Keywords:** dithiocarbamate-based organic polymer, vapor iodine capture, solution iodine capture, ultrafast removal, high uptake, regeneration

## Abstract

Organic polymers are widely explored due to their high stability, scalability, and more facile modification properties. We developed cost-effective dithiocarbamate-based organic polymers synthesized using diamides, carbon disulfide, and diamines to apply for environmental remediation. The sequestration of radioiodine is a serious concern to tackle when dealing with nuclear power for energy requirements. However, many of the current sorbents have the problem of slower adsorption for removing iodine. In this report, we discuss the utilization of an electron-rich dithiocarbamate-based organic polymer for the removal of iodine in a very short time and with high uptake. Our material showed 2.8 g/g uptake of vapor iodine in 1 h, 915.19 mg/g uptake of iodine from cyclohexane within 5 s, 93% removal of saturated iodine from water in 1 min, and 1250 mg/g uptake of triiodide ions from water within 30 s. To the best of our knowledge, the iodine capture was faster than previously observed for any existing material. The material was fully recyclable when applied for up to four cycles. Hence, this dithiocarbamate-based polymer can be a promising system for the fast removal of various forms of iodine and, thus, enhance environmental security.

## 1. Introduction

The challenge of meeting the energy requirement of the rapidly growing urbanized population is highly important. The deterioration of fossil fuel resources and increased carbon emissions requires an alternative energy resource to meet the needs. The world now looks forward to nuclear power as the energy source of the future as it emanates high energy output and a low carbon footprint [1,2]. However, the limitation lies in the discharge of volatile radioactive species, including different forms of iodine, while processing spent fuel [3,4]. Among those, ^129^iodine (I) is highly dangerous, with an extreme half-life period of more than one core years; it can emit harmful radiations and can cause deleterious effects once it enters the food chain. ^131^I possesses a shorter lifetime, is often used in radiation therapies, and is toxic to normal individuals; it is also expelled from nuclear power plants during processing and other unprecedented situations [5,6]. Conventionally, these iodine-containing compounds, including I_2_, are captured by acid/base treatment or by converting them into silver salts [7]. These techniques have shortcomings of high cost and the undesirable rerelease of iodine to the environment. Adsorption is now a widely accepted technique for iodine capture because of its lower cost, easier operation, and high efficiency.

Various materials have been reported to have iodine adsorption capacity, such as zeolites [8,9], ceramics [10], aerogels [11,12,13,14,15], metal–organic framework [15,16,17], covalent organic frameworks [18], activated carbon [19], and porous organic polymer [20,21,22,23]. High capture properties have also been shown by nonporous materials [24,25]. Porous organic polymers have been highly explored recently owing to their exceptional uptake capacity, high physicochemical stability, lower synthetic cost, and stronger linkages. These organic polymers have the viability to tune the skeletal structure according to the requirements. The iodine capture ability of the material depends on various properties such as porous surface area, extended conjugation, binding sites, and the heteroatoms present in the polymer structure [26]. Recently, more efforts have been executed to incorporate hetero atoms such as nitrogen, sulfur, oxygen, phosphorus, and boron to make the material electron-rich and, thus, obtain higher uptake [27]. The idea behind the incorporation of heteroatoms is that they turn the material into a Lewis base due to their electron-rich nature. This Lewis base can interact better with the Lewis acidic iodine, thereby enhancing the capture [28]. This is beneficial not only for higher uptake but also for the kinetics of the capture, which is exceedingly important for real applications [29].

Deliberating the importance of electron-rich organic polymer for efficiently capturing radioactive iodine, we synthesized a class of organic polymers (DTC-OP) with S, N, and O heteroatoms by incorporating dithiocarbamate and amide functional groups on the backbone. Dithiocarbamates are an important functionality for many materials and biomedical applications. However, no reports have ever mentioned the capability of dithiocarbamate systems for iodine capture, highlighting the novelty of this work. The synthesis of the material was straightforward and easy with readily available inexpensive starting materials and could be completed within a time frame of 2 h. These polymers were observed to capture molecular iodine in the vapor state, as well as from water and organic solvent; furthermore, they were able to remove aqueous I_3_^−^ with high uptake values. Even though there are reports of efficient materials with high iodine uptake [30], much less focus has been given to the time they take for the capture. Most of the material showed complete removal in several hours or even days, and this material showed purification in a few seconds for the first time to the best of our knowledge, reiterating this work’s novelty. It is also rare and ground-breaking to have a system that can take iodine from air, water, and organic solvent with consistently higher uptake in all cases and at a faster rate. Hence, our goal is to have a platform for radioactive iodine capture within a very short period with high uptake capacity. The experimental data indicate that the material is highly efficient in terms of uptake and time for the capture of iodine from various forms, and it can be further used as a solution for iodine contamination and storage. The material also has the perk of regeneration in that it can be used multiple times.

## 2. Results and Discussion

**Figure 1:**
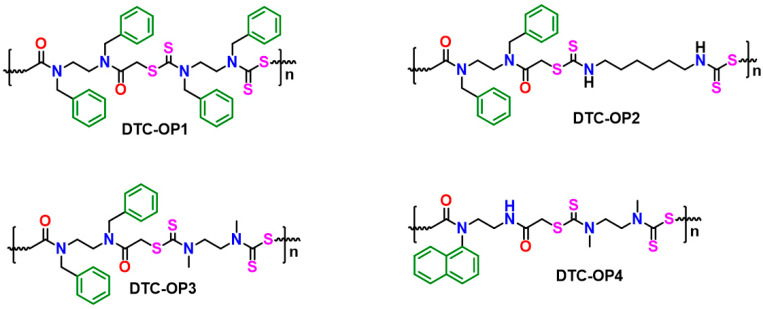
Structure of dithiocarbamate-based organic polymers (DTC-OP).

Organic polymers are known to be highly efficient materials for the capture of iodine and the removal of other environmental pollutants. We synthesized a class of dithiocarbamate-based organic polymer (DTC-OP) (Figure 1) with different functional groups and chain lengths, thus exhibiting different structural, porous, morphological, and mechanical properties. They were synthesized using a two-step reaction, where the first step was to convert functionalized diamine to diamide using chloroacetyl chloride. The polymerization was carried out via a multicomponent reaction of a functionally modified diamide, carbon disulfide, and another functionalized diamine of varying chain length. The reaction was fast and was completed within a short time of 15 min. The synthesis, properties, and characterization of the polymers were communicated in our previous report [31]. The design of the material was executed to be advantageous for iodine capture properties by adding heteroatoms such as N, S, and O. This was achieved by incorporating a dithiocarbamate group, amide functional group, and aromatics rings on its structure. Thus, this material is electron-rich and can effectively interact with various kinds of iodine. As the preliminary step to choose the best candidate among the four DTC-OPs, we performed a pilot experiment with iodine dissolved in cyclohexane. The removal efficiency was monitored by ultraviolet (UV)/visible spectrophotometry due to the decreased color of the highly colored iodine solution.

**Figure 2:**
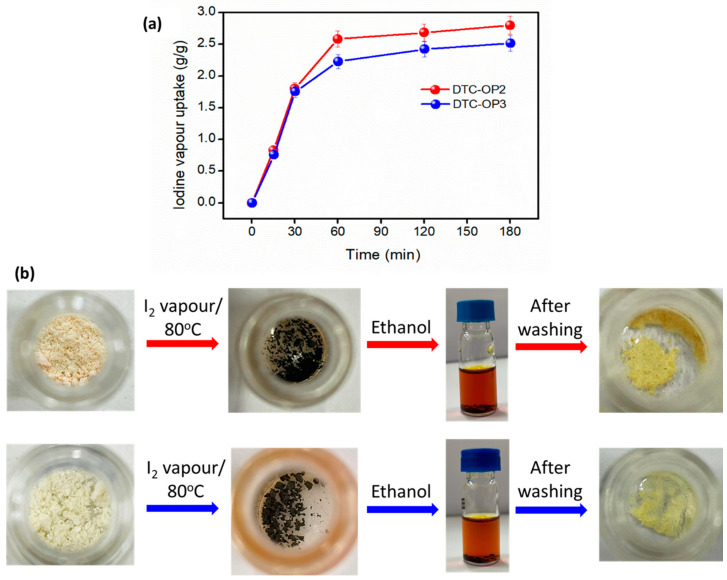
Adsorption of vapor iodine on DTC-OP2 and DTC-OP3. (**a**) Uptake of iodine on the material over different intervals of time. (**b**) Digital images of the material at various stages of iodine adsorption.

The iodine was adsorbed on all materials, and a comparison of UV/visible spectra suggested that DTC-OP2 and DTC-OP3 are potential candidates for iodine removal studies (Figure S1). Furthermore, all studies were carried out by these two candidates. The porosity studies of all the materials were carried out, and they exhibited hierarchical porosity. A pore size distribution of 3.114 nm and 2.508 nm, respectively, was shown for DTC-OP2 and DTC-OP3, higher than the pore size of DTC-OP1 and DTC-OP4. However, in comparison, the surface area was lower for DTC-OP2 and DTC-OP3 (27.198 m^2^/g and 10.406 m^2^/g), which commemorates the importance of pore size along with the surface area (Figures S2–S5 and Table S1). With these materials, all forms of iodine contaminations: air, aqueous (neutral and salt), and non-aqueous can be removed efficiently. The importance of iodine capture from various sources is portrayed in Figure 2.

### 2.1. Iodine Vapor Adsorption

Radioactive iodine is formed as the byproduct of uranium fuel used in nuclear reactors. The Chernobyl disaster was a devastating nuclear accident that occurred in 1986, and substantial radioactive iodine was expelled into the environment, taking many lives. The radioactive iodine ^131^I is very similar to the stable ^127^I, and the human body cannot distinguish between them. In effect, the thyroid gland stores this iodine and causes deleterious effects in the healthy human body. On account of their similarity, we chose the stable isotope ^127^I as the surrogate of the radioactive counterparts (^129^I and ^131^I). For studies of iodine vapor adsorption (gravimetrically), we heated the crystalline I_2_ at 80 °C so that enough vapors were evolved. The pre-weighed materials DTC-OP2 and DTC-OP3 were taken in weighed glass vials and kept inside an iodine-containing bottle, which was put in a hot air oven. The weight of the sample was noted at different intervals of time to monitor the progress of iodine uptake. A control experiment was also conducted with the same amount of material taken and heated at 80 °C. It was observed that that there was an increase in the weight of the sample kept in the iodine chamber as time progressed, which was an indication of the iodine vapor adsorption. The color of the material was also changed from beige to brownish black, denoting iodine uptake (Figure 2b). The uptake capacity of both DTC-OP2 and DTC-OP3 was calculated, and it was found that both showed passably high uptake capacities of 2.80 g/g and 2.51 g/g, respectively. The saturation in the uptake was observed within 60 min, which is very fast when compared to the state-of-the-art materials (Figure 2a). The release of adsorbed iodine was carried out by adding a polar solvent, ethanol. The adsorbed iodine was released into ethanol, giving a brown color to the solution, which was completed within 3 h (Figure S6). The material was regenerated after multiple washes with ethanol. 

**Figure 3:**
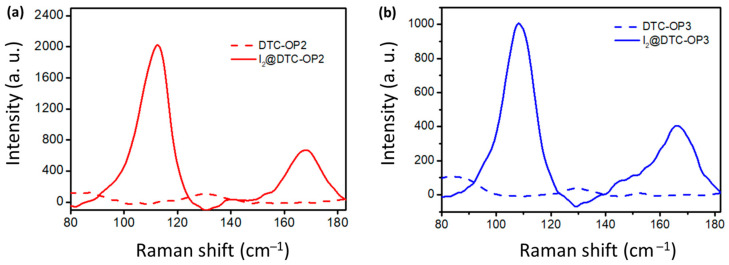
Raman spectra of (**a**) DTC-OP2 and (**b**) DTC-OP3 before and after iodine adsorption.

The iodine-adsorbed material was characterized using Raman spectroscopy. No peaks were observed in the Raman spectrum of DTC-POP2 and DTC-POP3, whereas two peaks appeared in the iodine-adsorbed material (Figure 3) at the vicinity of 169 cm^−1^ and 110 cm^−1^. The peak at 169 cm^−1^ is a characteristic stretching frequency (υ_I–I_) of iodine molecules. The prominent peak in the Raman spectra of both materials was at 110 cm^−1^ which is attributed to the symmetrical stretching mode of I_3_^−^ [32]. The antisymmetric stretching of I_3_^−^ should appear at 142 cm^−1^, as observed in the Raman spectra of both DTC-OP2 and DTC-OP3. From Figure 3, it is evident that the majority of I_2_ molecules were converted into I_3_^−^ [33]. The mechanism of iodine adsorption is also evident in the Raman spectrum. The electron-rich material can interact with the iodine molecule with a high affinity via a Lewis base–Lewis acid interaction concept. Thus, the lone pair of electrons in the non-bonding orbital (n) of N is donated to the antibonding orbital of iodine (σ*). This charged complex can attract more iodine molecules toward it and form I_3_^−^ [26,34]. 

### 2.2. Iodine Adsorption from Organic Solvent 

**Figure 4:**
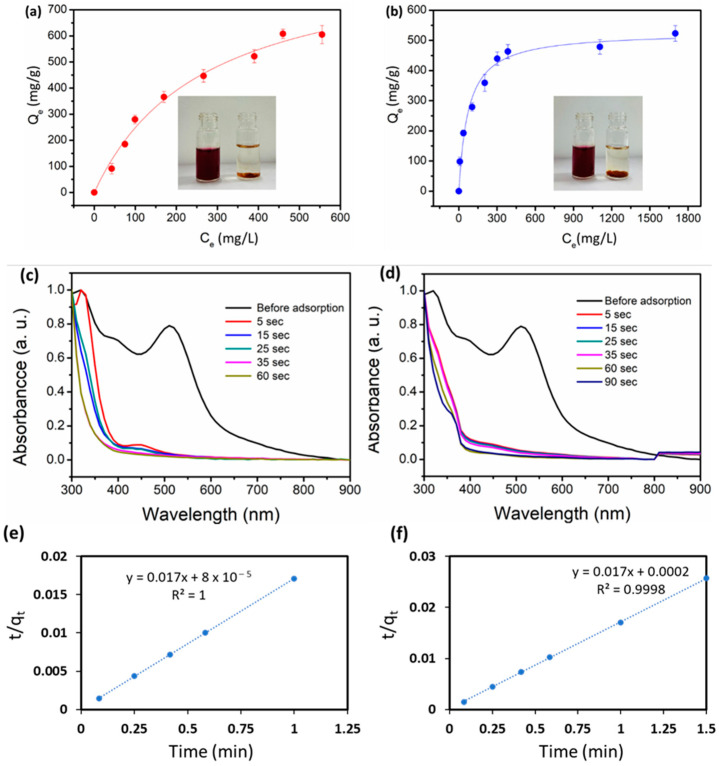
Iodine uptake from cyclohexane (fitted to Langmuir isotherm model) by (**a**) DTC-OP2 and (**b**) DTC-OP3. Inset: Photograph of iodine solution before and after adsorption with the iodine-adsorbed material settled down in the vial. Kinetic data of adsorption of iodine from cyclohexane by (**c**) DTC-OP2 and (**d**) DTC-OP3. Q_e_ is the Equilibrium uptake capacity and C_e_ is the equilibrium concentration of the iodine solution. (**e**) Pseudo-second-order rate kinetic adsorption fitting with DTC-POP2. (**f**) Pseudo-second-order rate kinetic adsorption fitting with DTC-POP3.

**Figure 5:**
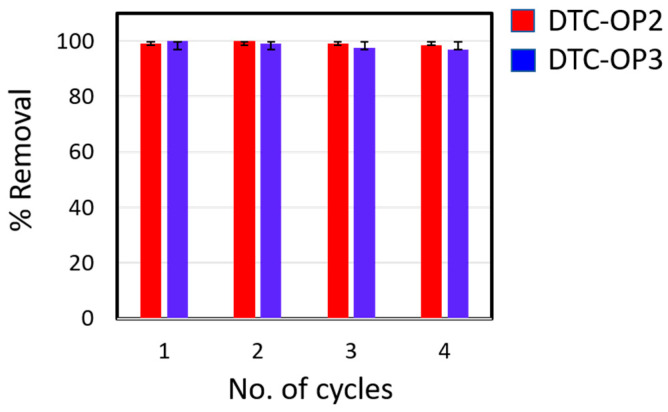
Reusability of the materials for the iodine capture.

The adsorption of iodine from the nonpolar organic solvent is also equally important for the capture of iodine from the waste effluents of nuclear reactors. To mimic that, iodine was dissolved in cyclohexane, which resulted in a deep purple color. The materials DTC-POP2 and DTC-POP3 were mixed with various concentrations of this solution. Immediate decolorization of the iodine solution was observed, and the progress of adsorption was monitored by UV/Vis spectroscopy. The adsorption isotherm was drawn, and the data were fitted well with the Langmuir adsorption model (Figure 4a,b). Uptake capacity was calculated, and it was found that DTC-OP2 showed a maximum uptake of 915.19 mg/g and DTC-OP3 showed a maximum uptake of 528.45 mg/g. A kinetics study was also carried out to further explore the rate of adsorption. To our surprise, it was observed that almost all iodine was removed completely within 5 s, as can be observed in the absorption spectrum in Figure 4c,d. The data were fitted well with the pseudo-second-order rate equation with rate constant values of 1.445 g/mg·min and 3.6125 g/mg·min, respectively, for DTC-OP2 and DTC-OP3 (Figure 4e,f). Both materials showed the fastest ever removal of iodine from the cyclohexane when compared to state-of-the-art materials. The regeneration of the iodine loaded on the material can be carried out by adding a polar solvent, for which we used ethanol. Complete regeneration of the material was observed when performed in four cycles; removal capacity was retained for up to four cycles without much loss in the activity (Figure 5). 

### 2.3. Iodine Adsorption from Aqueous Solution

**Figure 6:**
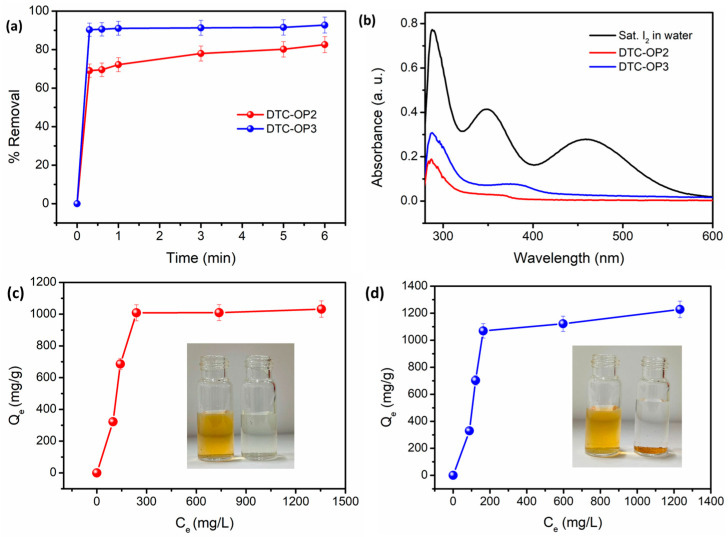
(**a**) Removal of saturated iodine from water over time with 10 mg of the material. (**b**) UV/Vis spectrum of I_2_ removal from water. Uptake of triiodide ions from water by (**c**) DTC-OP3 and (**d**) DTC-OP4. Q_e_ is the equilibrium uptake capacity and C_e_ is the equilibrium concentration of the iodine solution. Inset: Photograph of iodine solution before and after adsorption. The material at the bottom of the vial is iodine-adsorbed DTC-OP3.

Iodine can be present in water in two forms: iodine molecules and triiodide ions. Adsorption of saturated I_2_ and also I_3_^−^ was studied with both DTC-OP2 and DTC-OP3. The percentage removal of I_2_ from water over time was calculated by measuring the absorbance at regular intervals of time. It was observed that efficient removal was achieved by DTC-OP upon the interaction of iodine in water solution making the dark-yellow solution colorless. When both materials were compared, DTC-OP3 was found to be more efficient, with about 93% removal within 1 min, whereas only about 82% of removal was observed with DTC-OP2 within 6 min, and further increases were not observed as time progressed (Figure 6). 

**Figure 7:**
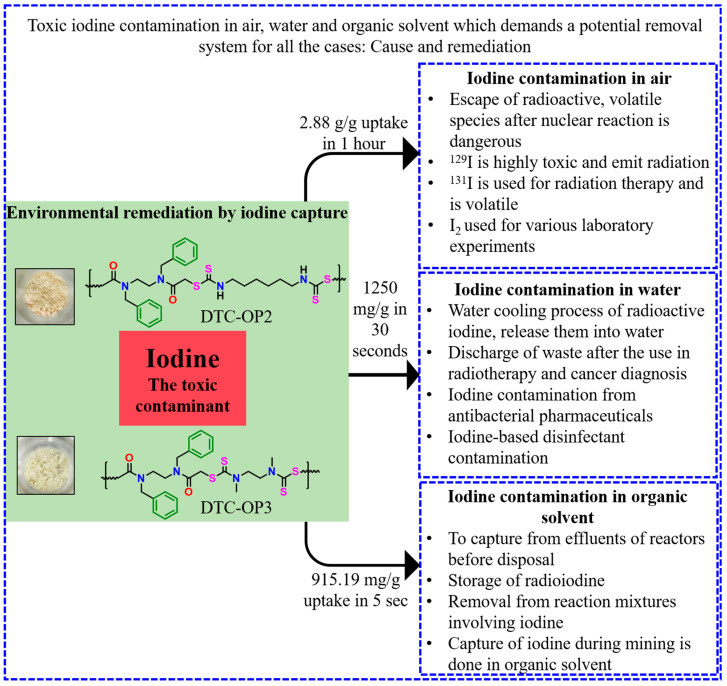
The importance of the capture of toxic iodine contaminants from air, water, and organic solvent by DTC-OP with the maximum uptake capacity and minimum time required.

Adsorption of triiodide ions from water by DTC-OP2 and DTC-OP3 showed that the removal was highly efficient, and we could observe a sudden color change of the triiodide solution when mixed with the material. Adsorption isotherms were drawn by carrying out the removal studies with various concentrations of the solution up to 2000 ppm. The data were fitted linearly with a Langmuir adsorption isotherm (Figure S7). A maximum uptake capacity of 1111.11 mg/g was shown by DTC-OP2, whereas DTC-OP3 had a higher uptake of 1250 mg/g. Kinetics studies were also carried out, and an excellent removal within a short time of 30 s (Figure S8) was observed. The influence of pH on the adsorption of iodine was also studied using DTC-OP2 and DTC-OP3. Immediate decolorization of the iodine solution was observed in all pH solutions (pH = 2, 4, 7, 9, and 11), thus showing a high percentage removal (Figure S9). These pH solutions were prepared by adding HCl and NaOH to get the desired pH. The results also deliver the information that the competing H^+^, Na^+^, Cl^−^, and OH^−^ did not affect the adsorption of the triiodide ions into the materials. The high uptake value and extremely high adsorption rate for the iodine capture make these materials highly competent for real-world applications (Figure 7). 

## 3. Materials and Methods

The synthesis of the DTC-OP was carried out in line with our previous report [31]. All chemicals and reagents were locally procured. They were analytically pure and used as is without purification. All absorbance measurements were taken using a Biotek Epoch 2 microplate reader with 96-well plates. Fourier-transform IR spectroscopy (FT-IR) was recorded in a Shimadzu IR Tracer 100 using the KBr pellet method. Raman spectroscopy was performed using a Horiba Labram HR Evo Raman Spectrometer. The spectra were plotted in OriginPro 8.5.1.

### 3.1. Selection of Best DTC-OP

First, 5 mg of each material, DTC-OP1 to DTC-OP4, was taken in separate vials, and 1 mL of 300 ppm of I_2_ in cyclohexane was added. The solution was passed through a 0.22 µm syringe filter, and the absorbance was measured. The percentage removal was calculated using the following equation [35]:

$$\%\;\mathrm{removal}=\frac{\left(C_{i}-C_{f}\right)\times 100}{C_{i}},$$

where *C_i_* is the initial concentration of the iodine solution before adsorption, and *C_f_* is the concentration after the adsorption. The same equation was used for all calculations of the percentage removal. 

### 3.2. Iodine Adsorption in Vapor Phase

First, 30 mg of the materials were taken in smaller vials, and the weight was carefully noted. These were kept in a bigger glass bottle containing a high quantity of crystalline iodine. The bottle with these vials were heated at 80 °C. At different intervals of time, the vials were taken out and kept at room temperature until reaching room temperature. The vials were properly cleaned, and their weights were measured. The weight percentage was calculated as follows [36]:

$$M=\frac{\left(m_{2}-m_{1}\right)\times 100}{m_{1}},$$

where *M* denotes the iodine uptake, and *m*_1_ and *m*_2_ represent the weight of the material before and after iodine adsorption. 

### 3.3. Iodine Release and Material Regeneration

First, 10 mg of the iodine loaded samples were taken in vials, before adding 1 mL of methanol each. A change in the color of methanol indicated the release of iodine. The amount of iodine released was calculated by measuring the absorbance at different intervals of time. The material was regenerated after multiple washes with methanol. 

### 3.4. Adsorption of Iodine in Liquid Phase

For the adsorption studies of iodine from cyclohexane, a stock solution of 2000 mg/L of iodine in cyclohexane was prepared. Other concentrations were prepared by diluting with cyclohexane. For the isotherm study, 5 mg of the material was taken in the vial, and 2 mL of iodine in cyclohexane of different concentrations was added. The solution was filtered using a 0.22 µm syringe filter, and the absorbance was measured to calculate the final concentration. The uptake capacity at the point was calculated as follows:

$$\mathrm{Uptake}\;\mathrm{capacity}=\frac{\left(C_{i}-C_{f}\right)\;V}{m},$$

where *C_i_* is the initial concentration of the solution before adsorption, *C_f_* is the concentration after adsorption, *V* is the volume of the iodine solution used in mL, and m is the mass of the adsorbent in mg. The adsorption isotherms were fitted using the Langmuir adsorption model as follows [37]:

$$\mathrm{Linear}\;\mathrm{fit}:\;\frac{C_{e}}{Q_{e}}=\frac{C_{e}}{Q_{m}}+\frac{1}{Q_{m}\;\times \;K_{l}},$$

$$\mathrm{Nonlinear}\;\mathrm{fit}:\;Qe=\frac{Q_{m}\;K_{l}\;C_{e}}{1+K_{l}\;C_{e}},$$

where *C_e_* is the final equilibrium concentration of the solution, *Q_e_* is the uptake capacity at the point, *Q_m_* is the maximum uptake capacity, and *K*_l_ is the Langmuir constant. 

The absorption study of iodine in aqueous solution was carried out by preparing a saturated solution of iodine in water, which was stirred overnight. I_3_^−^ adsorption studies were carried out using a solution made by dissolving I_2_ and potassium iodide (KI) in a 1:3 ratio and stirring overnight. Other concentrations were made from this stock solution. The amount of material and the volume of the solution used were the same as mentioned earlier. 

For the pH-dependent studies, 100 ppm solutions of I_3_^−^ were prepared in solutions of various pH (2, 4, 7, 9, and 11). These solutions were prepared by adding HCl or NaOH solution until the desired pH was obtained. For the adsorption studies, 1 mL of these 100 ppm solutions were added to 2 mg of DTC-OP2 and DTC-OP3.

### 3.5. Kinetics Studies

To 10 mg of material, 4 mL of iodine solutions (50 ppm of iodine in cyclohexane, 50 ppm triiodide in water, and saturated solutions of I_2_ in water) were added and stirred. Then, 400 µL of the solution was taken at different intervals of time, and the collected solutions were passed through the filter before measuring the absorbance. The rate of adsorption was calculated using the Ho–McKay model [38]:

$$\frac{t}{q_{t}}=\frac{1}{Kq^{2}}+\frac{t}{q},$$

where *t* is the time in min, *q_t_* (mg/g) is the uptake capacity at a particular time, *K* is the adsorption rate constant (g/mg·min), and *q* is the equilibrium adsorption capacity (mg/g). 

## 4. Conclusions

In summary, we developed a platform for the removal of various forms of iodine contamination from different sources by synthesizing an electron-rich polymer with dithiocarbamate and amide functional groups. These materials showed a high uptake of iodine vapor with more than 200 wt.% within a short time of 1 h. Similarly excellent capture properties were shown for iodine in cyclohexane, saturated iodine in water, and triiodide ions from the aqueous solutions. Even though the uptake capacity is comparable to several recently reported materials, our system excels in terms of kinetics with respect to the magical capture of iodine within a few seconds. These materials can also be regenerated and recycled several times. As the rate of adsorption is a very important parameter for a material to use for practical application, these DTC-OP materials are promising and competent candidates for iodine capture applications. It is also noteworthy that, as N- and S-rich carbonaceous materials are good applicants for iodine capture, dithiocarbamates represent a successful functional group for incorporation into the materials for such applications. We hope that the uptake capacity can be increased even further if a material can be synthesized with better textural properties such as a high surface area and pore volume by incorporating amides and dithiocarbamates into the backbone, which we are currently working on. Taken together, the new class of dithiocarbamate-based organic polymer reported here can be a potential material for the capture of radioactive iodine emitted from nuclear powerhouses, radioactive iodine used for medical treatments, and iodine-containing pharmaceuticals.

## Data Availability

Not applicable.

## Supplementary Materials

The supporting information can be downloaded at: https://www.mdpi.com/article/10.3390/ijms24021466/s1.

## References

[1] Adamantiades A., Kessides I. (2009). Nuclear Power for Sustainable Development: Current Status and Future Prospects. Energy Policy.

[2] Wang Q., Li R., He G. (2018). Research Status of Nuclear Power: A Review. Renew. Sustain. Energy Rev..

[3] Svensson P.H., Kloo L. (2003). Synthesis, Structure, and Bonding in Polyiodide and Metal Iodide− Iodine Systems. Chem. Rev..

[4] Nandanwar S.U., Coldsnow K., Utgikar V., Sabharwall P., Aston D.E. (2016). Capture of Harmful Radioactive Contaminants from Off-Gas Stream Using Porous Solid Sorbents for Clean Environment–A Review. Chem. Eng. J..

[5] Zhang Y., He L., Pan T., Xie J., Wu F., Dong X., Wang X., Chen L., Gong S., Liu W. (2022). Superior Iodine Uptake Capacity Enabled by an Open Metal-Sulfide Framework Composed of Three Types of Active Sites. CCS Chem..

[6] Xie L., Zheng Z., Lin Q., Zhou H., Ji X., Sessler J.L., Wang H. (2022). Calix [4] Pyrrole-based Crosslinked Polymer Networks for Highly Effective Iodine Adsorption from Water. Angew. Chem..

[7] Riley B.J., Vienna J.D., Strachan D.M., McCloy J.S., Jerden Jr J.L. (2016). Materials and Processes for the Effective Capture and Immobilization of Radioiodine: A Review. J. Nucl. Mater..

[8] Xu L., Li C.G., Zhang K., Wu P. (2014). Bifunctional Tandem Catalysis on Multilamellar Organic-Inorganic Hybrid Zeolites. ACS Catal..

[9] Zhou J., Lan T., Li T., Chen Q., Bai P., Liu F., Yuan Z., Zheng W., Luo X., Yan W. (2022). Highly Efficient Capture of Iodine in Spent Fuel Reprocessing Off-Gas by Novelly Porous Copper-Doped Silica Zeolites. Sep. Purif. Technol..

[10] Xiao H., Zhou H., Feng S., Gore D.B., Zhong Z., Xing W. (2021). In Situ Growth of Two-Dimensional ZIF-L Nanoflakes on Ceramic Membrane for Efficient Removal of Iodine. J. Memb. Sci..

[11] Gao R., Lu Y., Xiao S., Li J. (2017). Facile Fabrication of Nanofibrillated Chitin/Ag2O Heterostructured Aerogels with High Iodine Capture Efficiency. Sci. Rep..

[12] Hasanpour M., Hatami M. (2020). Photocatalytic Performance of Aerogels for Organic Dyes Removal from Wastewaters: Review Study. J. Mol. Liq..

[13] Katsoulidis A.P., He J., Kanatzidis M.G. (2012). Functional Monolithic Polymeric Organic Framework Aerogel as Reducing and Hosting Media for Ag Nanoparticles and Application in Capturing of Iodine Vapors. Chem. Mater..

[14] Lu Y., Liu H., Gao R., Xiao S., Zhang M., Yin Y., Wang S., Li J., Yang D. (2016). Coherent-Interface-Assembled Ag2O-Anchored Nanofibrillated Cellulose Porous Aerogels for Radioactive Iodine Capture. ACS Appl. Mater. Interfaces.

[15] Wu Y., Xie Y., Zhong F., Gao J., Yao J. (2020). Fabrication of Bimetallic Hofmann-Type Metal-Organic Frameworks@ Cellulose Aerogels for Efficient Iodine Capture. Microporous and Mesoporous Mater..

[16] Qasem K.M.A., Khan S., Ahamad M.N., Saleh H.A.M., Ahmad M., Shahid M. (2021). Radioactive Iodine Capture by Metal Organic Frameworks in Liquid and Vapour Phases: An Experimental, Kinetic and Mechanistic Study. J Environ. Chem. Eng..

[17] Xie W., Cui D., Zhang S.-R., Xu Y.-H., Jiang D.-L. (2019). Iodine Capture in Porous Organic Polymers and Metal–Organic Frameworks Materials. Mater. Horiz..

[18] Wang P., Xu Q., Li Z., Jiang W., Jiang Q., Jiang D. (2018). Exceptional Iodine Capture in 2D Covalent Organic Frameworks. Adv. Mater..

[19] Zhou J., Hao S., Gao L., Zhang Y. (2014). Study on Adsorption Performance of Coal Based Activated Carbon to Radioactive Iodine and Stable Iodine. Ann. Nucl. Energy.

[20] Guo Z., Sun P., Zhang X., Lin J., Shi T., Liu S., Sun A., Li Z. (2018). Amorphous Porous Organic Polymers Based on Schiff-Base Chemistry for Highly Efficient Iodine Capture. Chem.–Asian J..

[21] Mohan A., Al-Sayah M.H., Ahmed A., El-Kadri O.M. (2022). Triazine-Based Porous Organic Polymers for Reversible Capture of Iodine and Utilization in Antibacterial Application. Sci. Rep..

[22] Li Z., Li H., Wang D., Suwansoontorn A., Du G., Liu Z., Hasan M.M., Nagao Y. (2020). A Simple and Cost-Effective Synthesis of Ionic Porous Organic Polymers with Excellent Porosity for High Iodine Capture. Polymer (Guildf).

[23] Qian X., Wang B., Zhu Z.-Q., Sun H.-X., Ren F., Mu P., Ma C., Liang W.-D., Li A. (2017). Novel N-Rich Porous Organic Polymers with Extremely High Uptake for Capture and Reversible Storage of Volatile Iodine. J. Hazard. Mater..

[24] Jie K., Zhou Y., Li E., Li Z., Zhao R., Huang F. (2017). Reversible Iodine Capture by Nonporous Pillar [6] Arene Crystals. J. Am. Chem. Soc..

[25] Luo D., He Y., Tian J., Sessler J.L., Chi X. (2021). Reversible Iodine Capture by Nonporous Adaptive Crystals of a Bipyridine Cage. J. Am. Chem. Soc..

[26] Xiong S., Tang X., Pan C., Li L., Tang J., Yu G. (2019). Carbazole-Bearing Porous Organic Polymers with a Mulberry-like Morphology for Efficient Iodine Capture. ACS Appl. Mater. Interfaces.

[27] Pan X., Ding C., Zhang Z., Ke H., Cheng G. (2020). Functional Porous Organic Polymer with High S and N for Reversible Iodine Capture. Microporous and Mesoporous Mater..

[28] Pei C., Ben T., Xu S., Qiu S. (2014). Ultrahigh Iodine Adsorption in Porous Organic Frameworks. J. Mater. Chem. A Mater..

[29] Baig N., Shetty S., Pasha S.S., Pramanik S.K., Alameddine B. (2022). Copolymer Networks with Contorted Units and Highly Polar Groups for Ultra-Fast Selective Cationic Dye Adsorption and Iodine Uptake. Polymer (Guildf).

[30] Xie Y., Pan T., Lei Q., Chen C., Dong X., Yuan Y., Shen J., Cai Y., Zhou C., Pinnau I. (2021). Ionic Functionalization of Multivariate Covalent Organic Frameworks to Achieve an Exceptionally High Iodine-Capture Capacity. Angew. Chem. – Int. Ed..

[31] Thurakkal L., Porel M. (2023). Efficient Mercury Removal in 30 Seconds by Designing a Dithiocarbamate-Based Organic Polymer with Customizable Functionalities and Tuneable Properties. Environ. Sci. Water Res. Technol.

[32] Xie Y., Pan T., Lei Q., Chen C., Dong X., Yuan Y., Maksoud W.A., Zhao L., Cavallo L., Pinnau I. (2022). Efficient and Simultaneous Capture of Iodine and Methyl Iodide Achieved by a Covalent Organic Framework. Nat. Commun..

[33] Lin J.X., Liang J., Feng J.F., Karadeniz B., Lü J., Cao R. (2016). Iodine Uptake and Enhanced Electrical Conductivity in a Porous Coordination Polymer Based on Cucurbit [6] Uril. Inorg. Chem. Front..

[34] Harijan D.K.L., Chandra V., Yoon T., Kim K.S. (2018). Radioactive Iodine Capture and Storage from Water Using Magnetite Nanoparticles Encapsulated in Polypyrrole. J. Hazard. Mater..

[35] Mahanta D., Madras G., Radhakrishnan S., Patil S. (2008). Adsorption of Sulfonated Dyes by Polyaniline Emeraldine Salt and Its Kinetics. J. Phys. Chem. B.

[36] Abney C.W., Mayes R.T., Saito T., Dai S. (2017). Materials for the Recovery of Uranium from Seawater. Chem. Rev..

[37] García-Zubiri I.X., González-Gaitano G., Isasi J.R. (2009). Sorption Models in Cyclodextrin Polymers: Langmuir, Freundlich, and a Dual-Mode Approach. J. Colloid. Interface Sci..

[38] Ho Y.-S., McKay G. (1999). Pseudo-Second Order Model for Sorption Processes. Process biochem..

