# Supplementary material for: Superfast Capture of Iodine from Air, Water, and Organic Solvent by Potential Dithiocarbamate-Based Organic Polymer
Source: Int J Mol Sci. 2023 Jan 11;24(2):1466. doi: 10.3390/ijms24021466 (PMC9861013; doi:10.3390/ijms24021466)
Supplement: Supplementary file 1 [file ijms-24-01466-s001.zip › ijms-2091504-supplementary.pdf]

Superfast capturing of iodine from air, water, and organic solvent by potential dithiocarbamate-based organic polymer

Liya Thurakkal[1], Subba Rao Cheekatla[1] and Mintu Porel [1,2]*

[1]Department of Chemistry, Indian Institute of Technology Palakkad, Palakkad 678557, India

[2]Environmental Sciences and Sustainable Engineering Center, Indian Institute of Technology Palakkad, Palakkad 678557, India

*Correspondence: mintu@iitpkd.ac.in

Table of Contents

1. Figure S1. UV-visible spectra of I_2 in cyclohexane with the interaction of DTC-OPs……………………………………………………………………………………S2
2. Figure S2. a) Nitrogen sorption isotherm of DTC-OP1 at 77 K. The solid shapes represent adsorption, and the hollow shapes represent desorption. b) NLDFT pore size distribution of DTC-OP1…………………………………………………………...S2
3. Figure S3. a) Nitrogen sorption isotherm of DTC-OP2 at 77 K. The solid shapes represent adsorption, and the hollow shapes represent desorption. b) NLDFT pore size distribution of DTC-OP2…………………………………………………………S2
4. Figure S4. a) Nitrogen sorption isotherm of DTC-OP3 at 77 K. The solid shapes represent adsorption, and the hollow shapes represent desorption. b) NLDFT pore size distribution of DTC-OP3…………………………………………………………S3
5. Figure S5. a) Nitrogen sorption isotherm of DTC-OP4 at 77 K. The solid shapes represent adsorption, and the hollow shapes represent desorption. b) NLDFT pore size distribution of DTC-OP4…………………………………………………………S3
6. Table S1. Porosity properties of DTC-OP. (Specific surface area was calculated from the nitrogen adsorption isotherm using the BET method in the relative pressure (P/P_0) range from 0.1 to 0.3, pore volume was calculated at $P/P_0 = 0.99$)…………………S3
7. Figure S6. Release of adsorbed iodine to ethanol from (a) DTC-OP1 and (b) DTC-OP2……………………………………………………………………………………S4
8. Figure S7. Langmuir adsorption isotherm for the adsorption of triiodide ions from water by (a) DTC-OP2 and (b) DTC-OP3………………………………………S4
9. Figure S8. UV-vis spectrum for the kinetics of removal of triiodide ions from water by (a) DTC-OP2 and (b) DTC-OP3………………………………………………..S4
10. Figure S9. pH dependent studies on the adsorption of I_3^- from water by **(a)** DTC-OP2 **(b)** DTC-OP3 and **(c)** % removal at various pH……………………………………S5

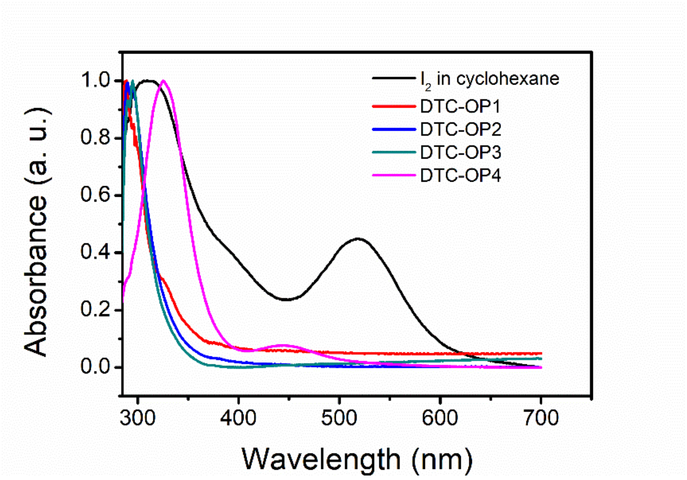

Figure S1. UV-visible spectra of I_2 in cyclohexane with the interaction of DTC-POPs.

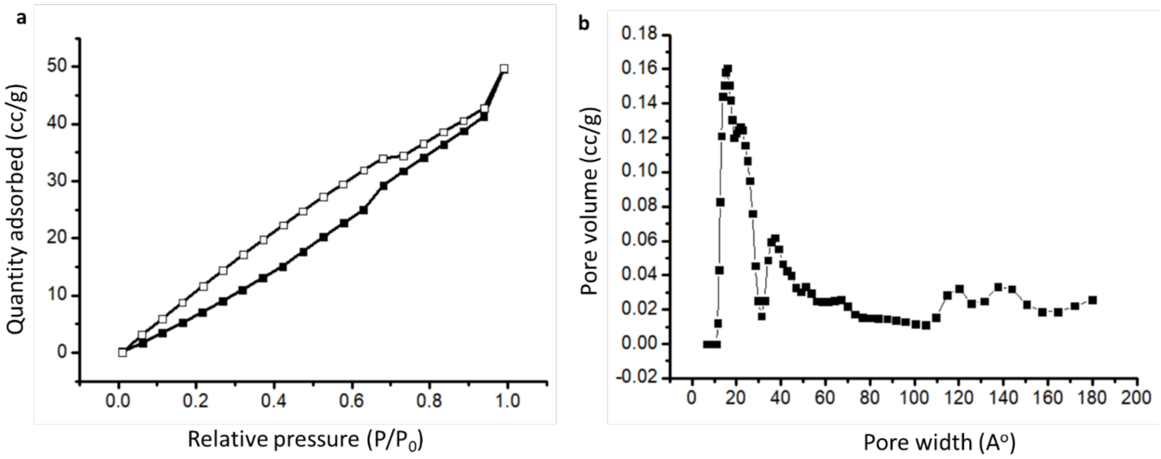

Figure S2. a) Nitrogen sorption isotherm of DTC-OP1 at 77 K. The solid shapes represent adsorption, and the hollow shapes represent desorption. **b)** NLDFT pore size distribution of DTC-OP1.

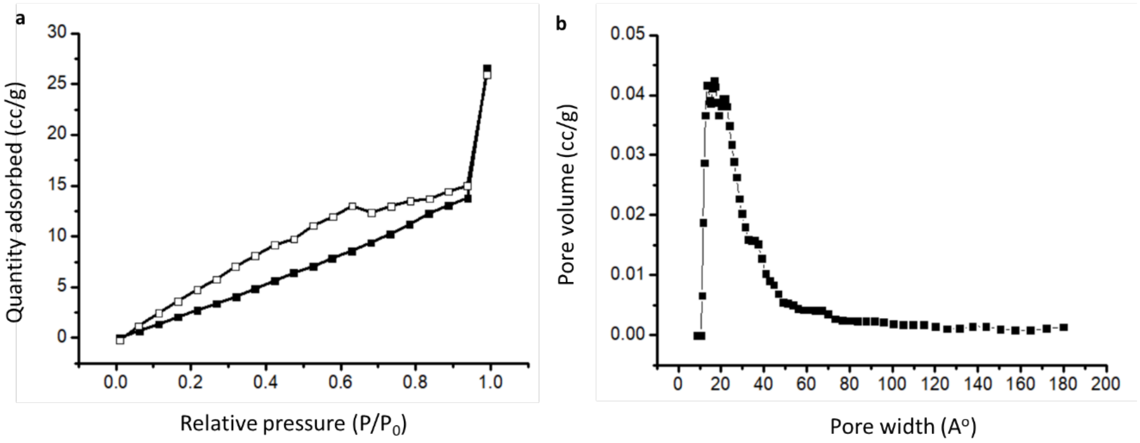

Figure S3. a) Nitrogen sorption isotherm of DTC-OP2 at 77 K. The solid shapes represent adsorption, and the hollow shapes represent desorption. **b)** NLDFT pore size distribution of DTC-OP2.

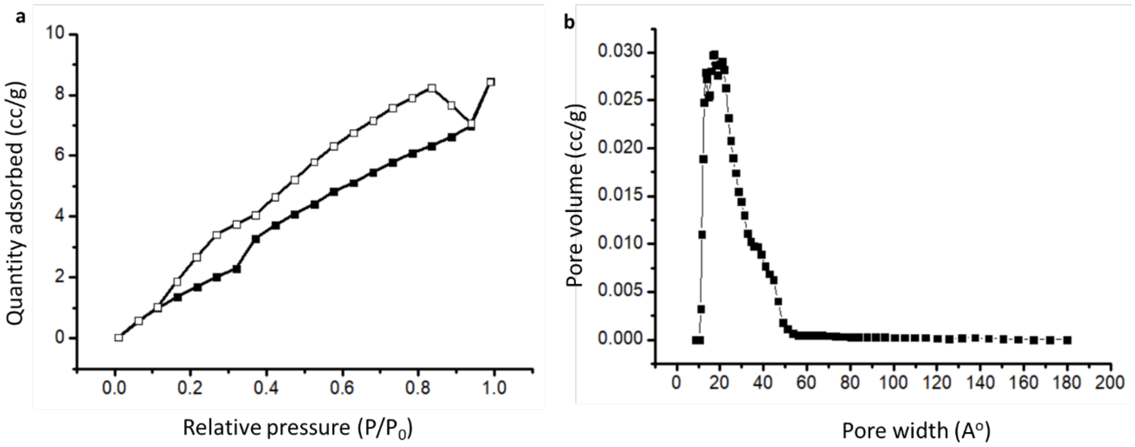

Figure S4. a) Nitrogen sorption isotherm of DTC-OP3 at 77 K. The solid shapes represent adsorption, and the hollow shapes represent desorption. **b)** NLDFT pore size distribution of DTC-OP3.

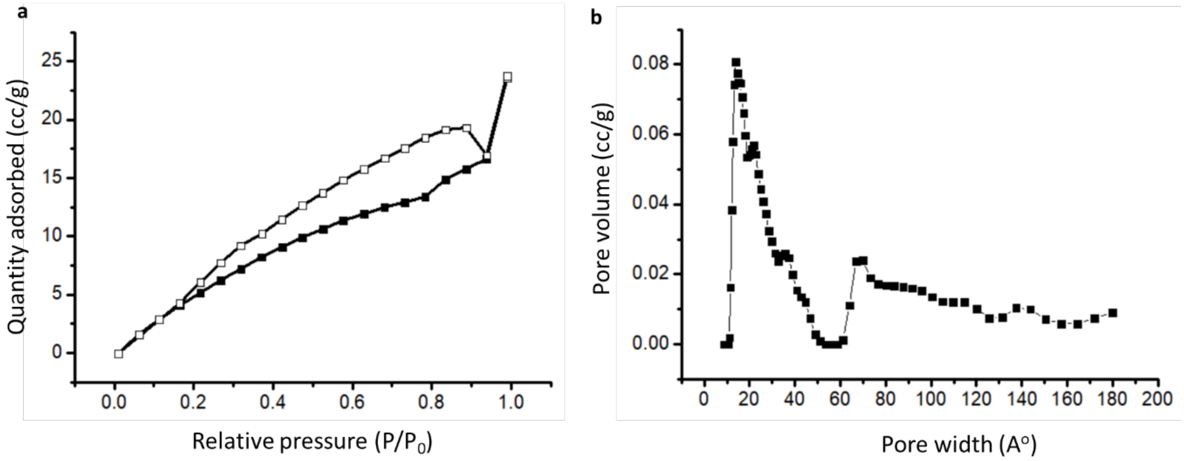

Figure S5. a) Nitrogen sorption isotherm of DTC-OP4 at 77 K. The solid shapes represent adsorption, and the hollow shapes represent desorption. **b)** NLDFT pore size distribution of DTC-OP4

Table S1. Porosity properties of DTC-OP. (Specific surface area was calculated from the nitrogen adsorption isotherm using the BET method in the relative pressure (P/P_0) range from 0.1 to 0.3, pore volume was calculated at $P/P_0 = 0.99$).

Material	BET surface area (m^2/g)	Pore volume (cc/g)	Pore size (nm)
**DTC-OP1**	90.119	0.0719	1.7055
**DTC-OP2**	27.198	0.0414	3.114
**DTC-OP3**	10.406	0.0137	2.508

**DTC-OP4**	34.476	0.0375	2.173

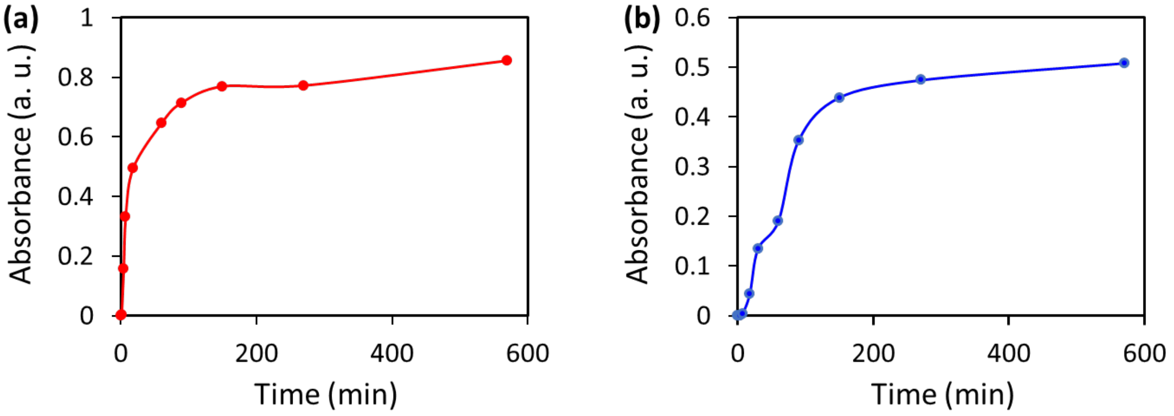

Figure S6. Release of adsorbed iodine to ethanol from **(a)** DTC-OP2 and **(b)** DTC-OP3

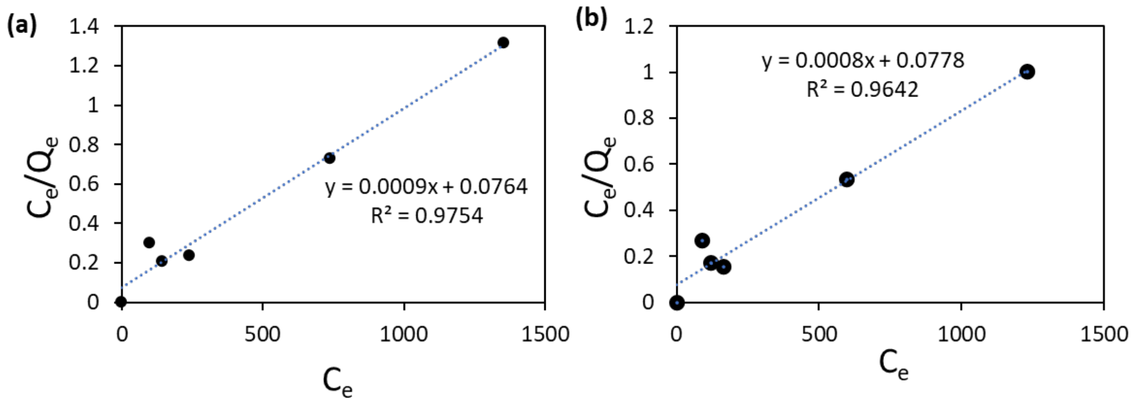

Figure S7. Langmuir adsorption isotherm for the adsorption of triiodide ions from water by **(a)** DTC-OP2 and **(b)** DTC-OP3

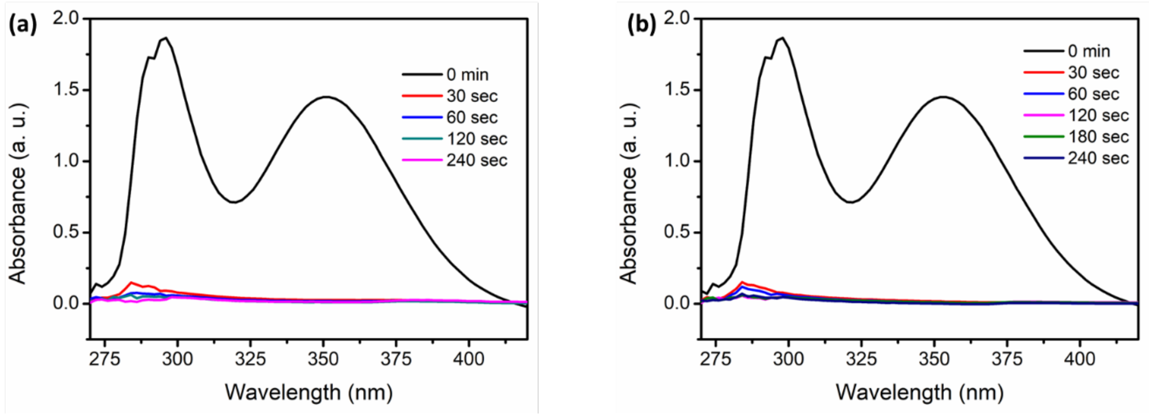

Figure S8. UV-vis spectrum for the kinetics of removal of triiodide ions from water by **(a)** DTC-OP2 and **(b)** DTC-OP3

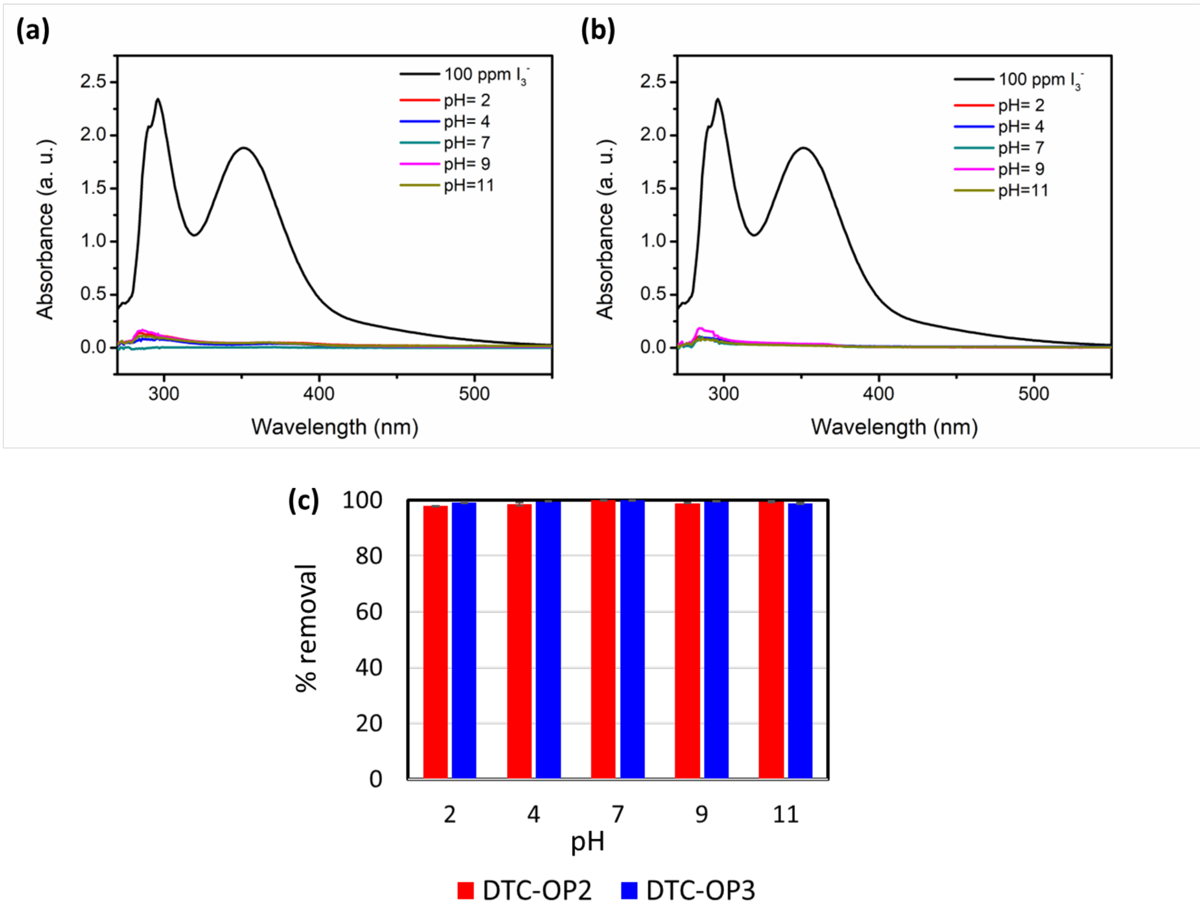

Figure S9. pH dependent studies on the adsorption of I_3^- from water by **(a)** DTC-OP2 **(b)** DTC-OP3 and **(c)** % removal at various pH
